# Case Report: Administration of Amniotic Fluid-Derived Nanoparticles in Three Severely Ill COVID-19 Patients

**DOI:** 10.3389/fmed.2021.583842

**Published:** 2021-03-17

**Authors:** Maria Ines Mitrani, Michael A. Bellio, Anthony Sagel, Marie Saylor, William Kapp, Kathryn VanOsdol, Gwendolyn Haskell, Danique Stewart, Zanub Abdullah, Ivan Santos, Julian Milberg, Alissa Arango, Albert Mitrani, George C. Shapiro

**Affiliations:** ^1^Organicell Regenerative Medicine, Inc., Miami, FL, United States; ^2^Landmark Hospital Athens, Athens, GA, United States; ^3^Landmark Hospital Naples, Naples, FL, United States; ^4^Technomad, Bonita Springs, FL, United States

**Keywords:** COVID-19, critical care, ARDS (acute respiratory distress syndrome), exosomes, amniotic fluid (AF), extracellular vesicles

## Abstract

**Rationale/Objectives:** A human coronavirus (HCoV-19) has caused the novel coronavirus disease (COVID-19) outbreak worldwide. There is an urgent need to develop new interventions to suppress the excessive immune response, protect alveolar function, and repair lung and systemic organ damage. Zofin (previously known as Organicell Flow) is a novel therapeutic that is derived from the soluble and nanoparticle fraction (extracellular vesicles and exosomes) of human amniotic fluid. Here within, we present the clinical outcomes after Zofin treatment in three critically ill patients suffering from severe, multi-organ complications induced by COVID-19 infection. All patients were diagnosed with COVID-19, developed respiratory failure, and were hospitalized for more than 40 days.

**Methods:** Zofin was administered to patients concurrently with ongoing medical care who were monitored for 28-days post-therapy. SOFA score assessment, chest X-rays, and inflammatory biomarker testing was performed.

**Main Results:** There were no adverse events associated with the therapy. The patients showed improvements in ICU clinical status and experienced respiratory improvements. Acute delirium experienced by patients completely resolved and inflammatory biomarkers improved.

**Conclusions:** Primary outcomes demonstrate the therapy was safe, accessible, and feasible. This is the first demonstration of human amniotic fluid-derived nanoparticles as a safe and potentially efficacious therapeutic treatment for respiratory failure induced by COVID-19 infection.

## Background

A human coronavirus (HCoV-19) has caused the novel coronavirus disease (COVID-19) outbreak worldwide. The main symptoms of COVID-19 include fever, fatigue, and cough that can progress rapidly to severe and critical conditions resulting in pulmonary edema leading to the most common complications: acute lung injury (ALI) and acute respiratory distress syndromes (ARDS) (1–5). The majority of cases result in mild symptoms, but some can progress into pneumonia and multi-organ failure. According to severity, it is divided into mild, normal, severe, and critically ill, the last of which is associated with ICU admission and mortality (6). Immune activation in some patients and the appearance of cytokine storm syndrome is one of the most potent causes of severe damage to lungs and other organs, which may lead to death.

In the early stages of the COVID-19 pandemic, treatment for COVID-19 patients included non-specific anti-viral, anti-inflammatory medication, oxygen therapy, mechanical ventilation, and blood pressure medications. A definitive antiviral therapy or ALI treatment for patients with or recovering from COVID-19 infections continues to be in development. Furthermore, as the number of COVID-19 infections continues to rise, severe cases continue to result in organ failure and long-term impairments. As a result, there remains an urgent need to develop new interventions to suppress the excessive immune response in a timely manner during the course of disease, protect alveolar function, and repair the pulmonary and systemic organ damage caused after the infection (7).

One hallmark feature of critical COVID-19 patients is the extremely high expression of pro-inflammatory markers, including C Reactive protein (CRP) and cytokines IL-6, TNFα, and IL8 (8). Anti-inflammatory and immune modulatory therapies have risen as strong therapeutic candidates because inflammatory cytokine storm is common in severe cases and is the highest amongst non-survival patients (8). Exaggerated immune response, the secretion of pro-inflammatory cytokines, and marked pulmonary infiltration are lethal components of viral infections (9). Therefore, traditional anti-inflammatory treatments, such as the anti-interlukin-6 receptor monoclonal antibody Tocilizumab and corticosteroids, are actively being used to suppress cytokine storms and prevent further injury. However, current standard of care anti-inflammatory medications are not optimal due to the concern that they may delay the elimination of the virus and risk secondary infection (8). Furthermore, severe COVID-19 cases continue to persist despite the incorporation of these current treatment options. Cell-based therapies are actively being tested for COVID-19 infections due to the observed immunomodulatory and anti-inflammatory effects (10). However, cell therapies are often limited due to cell delivery challenges and inadequate cell survival post-infusion (11). Furthermore, research has uncovered that cell-beneficial effects are mainly paracrine-mediated via the release of growth factors, cytokines, and extracellular vesicles such as exosomes rather than engraftment and differentiation (12, 13). Therefore, our proposed therapeutic intervention utilizes cell- and tissue-secreted paracrine factors, rather than the cells themselves, as the active drug components.

Zofin (previously known as Organicell Flow) is a novel biologic that is derived from the soluble and nanoparticle fraction of human amniotic fluid. The manufactured Zofin therapeutic is an acellular product characterized to contain over 300 growth factors, cytokines, and chemokines as well as other extracellular vesicles and exosomes secreted from perinatal tissues. Amniotic fluid sourced biologics have been studied for the therapeutic use of orthopedic repair due to the abundance of anti-inflammatory and tissue healing components (14–16). However, the use of amniotic fluid-derived paracrine factors, including proteins and nanoparticles, have not been tested for the treatment of COVID-19-induced systemic organ damage and respiratory failure.

Here within, we present three case reports of critically ill patients suffering from severe, multi-organ complications induced by COVID-19 infection who were treated with Zofin in addition to the authorized standard of care available at that time. These patients represent the subset of COVID-19 patients most effected by the virus. These three patients tested positive for severe acute respiratory syndrome coronavirus 2 (SARS-CoV-2) using a real-time reverse transcriptase polymerase chain reaction assay, were diagnosed with ARDS, and suffered from severe organ damage and respiratory failure. At the time of hospital admission there was no emergency authorized standard of care for COVID-19 infection. These patients were treated with supplemental oxygen, anti-inflammatories, antibiotics, antiviral medication, and other medications required to manage their multiorgan failure symptoms. Prior to enrollment for investigational treatment at the study site ICU (Landmark Hospital of Athens), these patients had been hospitalized for over 40 days and were transferred from the initial hospital sites to appropriately manage multiple comorbidities. The physicians explored all authorized pharmacological options available to them at that time. Due to the concern for declining and irreversible injury, the treating physician requested single patient emergency, compassionate use IND (eIND) to administer Zofin.

The first patient (de-identified subject CU#1) approved for Zofin was a 74-year-old Caucasian female with multiple comorbidities including obesity, hypertension (HTN), type 2 diabetes, depression, hyperlipidemia (HLD), and vitamin D deficiency. This patient was initially admitted to the hospital 44 days prior to treatment at the study site. Initial diagnosis was acute hypoxemic respiratory failure with positive COVID-19 infection. The patient was orally intubated after 6 days in the hospital and treated for COVID-19 with pneumonia. COVID-19+ tests were reported for 20 days post-admission. Tracheostomy placement was performed prior to transfer to the study site. Patient CU#1 continued to require mechanical ventilation prior to treatment and developed acute metabolic encephalopathy with ICU delirium along with acute kidney injury and anemia. The initial COVID-19 treatment upon admission included a 10-day course of Hydroxychloroquine, three doses of Ribavarin, and Kaletra. Inclusion of additional medication was ongoing to manage complications induced by the multiple comorbidities. Ten days after transfer to the study site, Zofin infusion was initiated in addition to ongoing medical treatment. Due to the patient's high BMI, it was decided to administer a total of four doses instead of the originally planned three. FDA approval was obtained prior to this protocol change.

The second patient (de-identified subject CU#2) was a 79-year-old Caucasian female with multiple chronic comorbidities including obesity, HTN, HLD, Hodgkin's disease, hypothyroidism (HYT), and status post-left carotid endarterectomy. This patient was admitted to the hospital 47 days prior to treatment at the study site. Her initial diagnosis included septic syndrome and hypoxemic respiratory failure with positive COVID-19 infection. The initial treatment reports did not indicate if this patient received any anti-viral medication to target the COVID-19 infection; instead, the treatment included a wide range of medications to manage the severe symptoms associated with comorbidities, antibiotics (Cefepime and Vancomycin), and hemodialysis. Due to the severity of the patient upon admission, mechanical ventilation was immediately required after 2 days. The patient was extubated and then reintubated, followed by tracheostomy placement. COVID-19+ tests were reported for 16 days post-admission. Hospital course was complicated by acute kidney injury, anemia requiring blood transfusion, encephalopathy, and septic shock. The patient underwent PEG placement a few days prior to transfer to the study site. Transfer of the patient to the study site was completed for ventilator liberation and management of other comorbidities. Like the previous patient, the high BMI of CU#2 qualified this patient for four doses of Zofin, as approved by the FDA.

The third patient (de-identified subject CU#3) was a 66-year-old Hispanic male with comorbidities that included type 2 diabetes and HTN. This patient was admitted to the hospital 42 days prior to treatment at the study site. Initial diagnosis was hypoxemic respiratory failure secondary to COVID-19 pneumonia. Initial COVID-19 treatments included Hydroxychloroquine as an outpatient, followed by Tocilizumab on admission. Patient CU#3 required intubation that was followed by extubation and reintubation. Hospital course was complicated by hypoxemic cardiac arrest, acute kidney injury that required renal replacement therapy, acute DVT, and encephalopathy. The patient underwent tracheostomy prior to transfer to the study site. Transfer to the study site was completed for ventilator liberation, management of hemodialysis, continuation of nutritional support, and management of other comorbidities. COVID-19+ tests were reported for 8 days post-admission. Additional medication and antibiotics were incorporated throughout treatment as the patient's condition evolved. The patient received three doses of Zofin beginning 9 days after transfer to the study site.

The primary objective of these three eINDs was to demonstrate safety, feasibility, and accessibility of Zofin for the treatment of these severely ill patients. Secondarily, we aimed to observe post-treatment changes in clinical status improvement and inflammatory biomarker improvement that may suggest potential therapeutic efficacy. Our underlying hypothesis suspected that Zofin treatment would improve patient outcomes and promote lung and organ failure assessment recovery. This report demonstrates the first use of an amniotic fluid-derived product in humans as a potential therapeutic to aid in the recovery from severe organ injuries induced by COVID-19 infection.

## Methods

### Ethics

This study involving human participants was reviewed and approved by the FDA and the Independent Review Board Western Institutional Review Board. The patient's consent to participate in the study and for data publication was obtained at the treatment site by the patient's proxy using an IRB approved informed consent form. These single patient INDs were submitted under the approved parent IND #19881 and FDA approval was issued with the following IND numbers: CU#1 IND#22370, CU#2 IND#22371, and CU#3 IND#22897. IRB approval was issued by letters of acknowledgment from the Western Institutional Review Board.

### Therapeutic Intervention

The therapeutic intervention studied in these case reports was an acellular biologic called Zofin. Zofin was manufactured by Organicell Regenerative Medicine, Inc. in Miami, FL. The Zofin product was derived from human amniotic fluid donated from consenting adults during routine, planned cesarean sections under IRB approved donor screening (IRB approval agency: IRCM). Donor qualification was performed under FDA CFR 1271. Donor qualification was certified following the review of the mother's medical history, social history, physical examination, and raw product recovery information. Relevant communicable disease testing was completed, and the mother was reported to have negative/non-reactive results for CMV total Ab, Hepatitis B core total Ab, Hepatitis B surface Ag, Hepatitis C virus Ab, HIV-1/HIV-2 Plus O, HTLV I/II Ab, Syphilis screening—non-treponemal, Ultrio Elite HBV, Ultrio Elite HCV, Ultrio Eliter HIV-1/2, and WNV. Upon receipt, the collected amniotic fluid was subjected to centrifugation and proprietary filtration to remove large particle debris and preserve the natural protein, nanoparticle, and exosome composition of amniotic fluid. The final Zofin product was released by Organicell Regenerative Medicine, Inc. after meeting the release criteria requirements. The specific release criteria parameters for the product administered in these treatments were: sterility (14-day cultures: no growth for aerobic, anaerobic, and fungal contamination), endotoxin (<0.05 EU/mL), nanoparticle composition (concentration = 3.26 × 10^11^/mL, mode particle size = 90.2 nm), protein concentration (2.83 mg/mL), and hyaluronic acid concentration (261 ng/mL). Zofin was stored frozen and shipped on dry ice to the treatment location following validated storage and shipping methods.

### Patient Standard Care and Administration of Therapeutic Intervention

Patient care and product infusion throughout the study period was performed at Landmark Hospital Athens, in Athens, GA. Standard care was followed along with Zofin administration under approved single patient eIND by the FDA and under IRB oversight. The patient care was defined by the treating physician in accordance with the authorized standard of care practices ongoing at the study site at the time of patient enrollment. The care was focused on treating the multiple ongoing organ failure issues induced by the COVID-19 infection. At the time of hospital admission, there was no standardized or authorized therapy for COVID-19 infection. These patients were treated with available anti-viral, anti-inflammatory, antibiotics, and other medication required to manage their occurring symptoms. Supplemental oxygen therapy and ventilation was provided as determined by the physicians. Zofin was administered intravenously at a dose of 1 mL diluted in 100 mL of normal saline. Intravenous infusion was performed at a rate of 2 mL/min. Product thawing and dilution occurred immediately before administration. Patients CU#1 and CU#2 received a total of four doses administered on day 0, day 4, day 6, and day 8. Patient CU#3 received a total of three doses administered on day 0, day 4, and day 8.

### SOFA Score Assessment

ChartPad Software (Technomad), a cloud-based electronic data capture platform, was used to collect patient data. SOFA score was calculated as reported in the literature (17) and was assessed on 0, 4, 6, 8, 14, 21, and 28 days after the initiation of Zofin therapy. The SOFA score was derived from clinical and laboratory results obtained for respiration (PaO_2_/FiO_2_, mmHg), coagulation (platelets, × 10^3^/μL), liver (bilirubin, mg/dL), cardiovascular (mean arterial pressure), neurologic (Glasgow coma score), and renal (creatinine, mg/dL).

### Chest X-Ray

A portable chest x-ray (CXR) was used to acquire imaging at baseline and throughout treatment to evaluate, identify, and monitor lung abnormalities (18). After images were acquired, analysis was performed by the radiologist at Landmark Hospital and CXR reports were generated to outline the clinical findings.

### Biomarker Testing

Biomarker collection occurred at 0, 4, 6, 8, 14, 21, and 28 days after initiation of Zofin therapy to assess for concentration of D-Dimer, CRP, IL2, IL6, and TNFα. D-Dimer and CRP measurements were performed at the Athens Regional Labs, while IL2, IL6, and TNFα were measured by Quest Diagnostics.

## Results

### Follow up of ICU Clinical Status

The clinical status of the patients was monitored for 28 days post-initiation of Zofin therapy. After treatment, the respiratory status of the patients improved and stabilized. CU#1 respiratory status improved throughout the 28 days, changing from a 21% oxygen T-collar to room air with no oxygen therapy requirement, CU#2 respiratory status improved during the 28 days with a transition from mechanical ventilation to non-mechanical ventilation, and the respiratory status of CU#3 improved 4 days post-treatment with decannulation and subsequent removal from oxygen therapy by day 14. Furthermore, all patients were transferred out of ICU status to the step-down unit within the 28-day period (Table 1). Patient CU#1 was discharged from the hospital 29 days post-treatment initiation and patient CU#3 was discharged from the hospital 26 days post-treatment initiation. Patient CU#2 remained in the hospital as she had experienced setbacks due to aspiration but was controlled and stable in the step-down unit with ventilation and hemodialysis.

**Table 1:** Clinical status of compassionate use patients.

**Patient ID**	**CU#1**	**CU#2**	**CU#3**
Age	74	79	66
Gender	F	F	M
Weight (kg)	118.75	100.24	60.36
BMI (kg/m^2^)	38.43	40.18	22.14
Prior comorbidities	Obesity, HTN, T2DM, depression, HLD, Vit. D deficiency	Obesity, HTN, HLD, Hodgkin's disease, HYT, status post left carotid endarterectomy	T2DM, HTN
Pre-treatment complications	Hyperglycemia Acute lung injury (ARDS) Anemia with normocytic indices	HyperglycemiaAcute renal failureAnemia requiring blood transfusionAcute lung injury (ARDS)	Acute renal injury Acute renal failure Acute lung injury (ARDS) Hyperglycemia
Days hospitalized Prior to treatment	44	48	42
Respiratory status	Base: 21% T-Collar Day 4: 21% T-Collar Day 6: 21% T-Collar Day 8: 21% T-Collar Day 14: 21% T-Collar Day 21: PMV room air Day 28: room air	Base: CPAP 5 PS 10 30% Day 4: CPAP 5 PS 10 35%. Weaned PS to 8 and FiO_2_ to 30% Day 6: CPAP PS 40 % Day 8: Patient placed on 40% ATC at 0811 and the PMV at 1531 Day 14: Patient placed on 40% ATC at 0811 and the PMV at 1531 Day 21: 40% T-Collar Day 28: 30% T-Collar	Base: PS/CPAP 8/5 24% Day 4: Patient tolerates trial cap and is successfully decannulated Day 8: Placed on 2L of O2 Day14: Weaned down to room air Day 21: Remains in room air Day 28: Discharged 26 days after baseline
Status after 28-day follow up period	Decannulated, discharged	Step-down unit, remained in ventilation and hemodialysis	Decannulated, not in hemodialysis, discharged

*HTN, hypertension; T2DM, type-2 diabetes; HLD, hyperlipidemia; HYT, hypothyroidism*.

### Effect of Zofin on SOFA Score

Improvement in SOFA score was found in all patients. SOFA score calculations decreased from 3 to 0 in CU#1 within 28 days, from 7 to 4 in CU#2 within 28 days, and from 4 to 0 in CU#3 within 21 days (Table 2). Assessment of the individual parameters used to calculate SOFA score showed improvements in PaO_2_/FiO_2_ and Glasgow score for CU#1, improvements in Glasgow score and creatinine levels for CU#2, and improvements in PaO_2_/FiO_2_ and creatinine levels for CU#3 (Table 2). Platelet count, bilirubin, and MAP measurements remained stable throughout the treatment course.

**Table 2:** SOFA score parameters.

**Patient**	**SOFA Score**	**PaO_**2**_/FiO_**2**_**	**Platelet Count (× 10^**3**^)**	**Bilirubin (mg/dL)**	**Glasgow score**	**MAP(mmHg)**	**Creatinine (mg/dL)**
CU#1	Day 0: 3	Day 0: 342	Day 0: 310	Day 0: 0.5	Day 0: 10–12	Day 0: 77–103	Day 0: 0.78
	Day 4: 2	Day 4: 457	Day 4: 364	Day 4: 0.7	Day 4: 10–12	Day 4: 65–86	Day 4: 1.10
	Day 6: 1	Day 6: 466	Day 6: 340	Day 6: 0.6	Day 6: 13–14	Day 6: 92–96	Day 6: 0.89
	Day 8: 1	Day 8: 476	Day 8: 329	Day 8: 0.6	Day 8: 13–14	Day 8: 74–94	Day 8: 0.98
	Day 14: 0	Day 14: 462	Day 14: 405	Day 14: 0.6	Day 14: 15	Day 14: 79	Day 14: 0.89
	Day 21: 0	Day 21: 471	Day 21: 423	Day 21: 0.7	Day 21: 15	Day 21: 75	Day 21: 0.94
	Day 28: 0	Day 28: 471	Day 28: 342	Day 28: 0.6	Day 28: 15	Day 28: 95	Day 28: 0.97
CU#2	Day 0: 7	Day 0: 242	Day 0: 183	Day 0: 0.4	Day 0: 10–12	Day 0: 62–94	Day 0: 3.55
	Day 4: 7	Day 4: 268	Day 4: 204	Day 4: 0.3	Day 4: 10–12	Day 4: 76–92	Day 4: 3.74
	Day 6: 4	Day 6: 280	Day 6: 190	Day 6: 0.5	Day 6: 15	Day 6: 85–95	Day 6: 1.98
	Day 8: 3	Day 8: 232	Day 8: 201	Day 8: 0.4	Day 8: 15	Day 8: 73–96	Day 8: 1.79
	Day 14: 4	Day 14: 245	Day 14: 214	Day 14: 0.3	Day 14: 15	Day 14: 93	Day 14: 3.28
	Day 21: 4	Day 21: 240	Day 21: 201	Day 21: 0.3	Day 21: 15	Day 21: 82	Day 21: 2.29
	Day 28: 4	Day 28: 271	Day 28: 269	Day 28: 0.3	Day 28: 15	Day 28: >70	Day 28: 2.27
CU#3	Day 0: 4	Day 0: 350	Day 0: 428	Day 0: 0.3	Day 0: 15	Day 0: 90	Day 0: 3.96
	Day 4: 3	Day 4: 466	Day 4: 540	Day 4: 0.3	Day 4: 15	Day 4: 89	Day 4: 4.16
	Day 6: –	Day 6: –	Day 6: –	Day 6: –	Day 6: –	Day 6: –	Day 6: –
	Day 8: 3	Day 8: 471	Day 8: 723	Day 8: 0.6	Day 8: 15	Day 8: 99	Day 8: 5.2
	Day 14: 2	Day 14: 471	Day 14: 507	Day 14: 0.3	Day 14: 15	Day 14: 85	Day 14: 2.73
	Day 21: 0	Day 21: 467	Day 21: 294	Day 21: 0.3	Day 21: 15	Day 21: 84	Day 21: 1.24
	Day 28: –	Day 28: –	Day 28: –	Day 28: –	Day 28: –	Day 28: –	Day 28: –

–*, sample not taken*.

### Effect of Zofin on Lung Imaging

**Figure 1:**
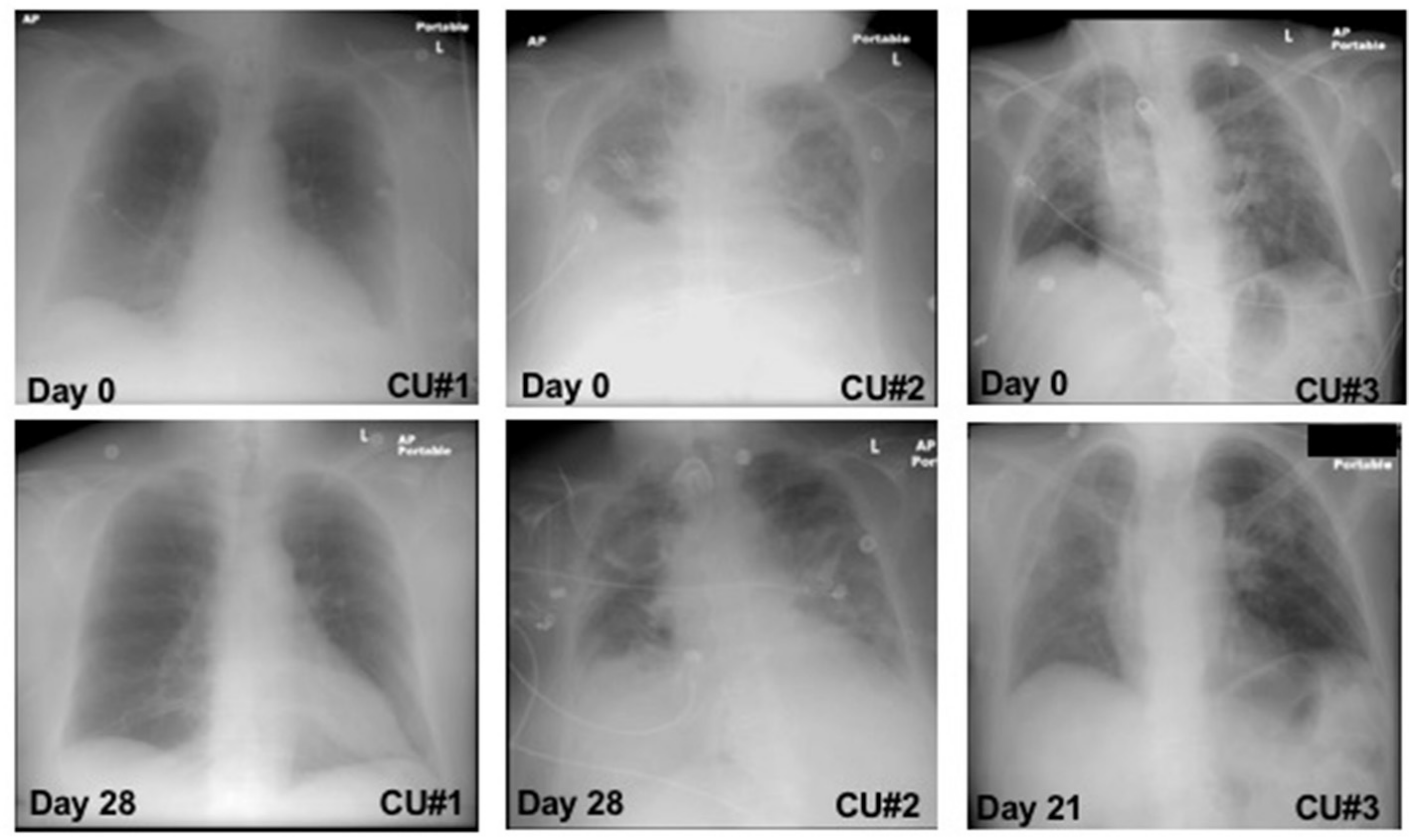
Chest-X ray images. Chest-X ray images of patient CU#1 at day 0 and day 28 (Left). Chest-X ray images of patient CU#2 at day 0 and day 28 (Middle). Chest-X ray images of patient CU#3 at day 0 and day 21 (Right).

CXR images were collected throughout the treatment and the changes from baseline to day 21 and 28 were observed and reported (Figure 1). CXR analysis of patient CU#1 displayed at baseline show an infiltrate present in the left lower lobe with no defined pleural fluid. After 28 days, CXR showed basilar, infrahilar air space opacity present bilaterally in the base of the lungs. CXR analysis of patient CU#2 displayed bilateral pulmonary disease at baseline. At day 28, CXR analysis showed small pleural effusions. CXR analysis of patient CU#3 displayed bilateral upper lobe infiltrate at baseline. At day 21, CXR analysis showed residual consolidation present in the left perihilar region. There was partial interval resolution of right upper lobe pneumonic consolidation.

### Inflammatory Biomarker Assessment

Quantification of inflammatory biomarkers was completed at each testing time point (Table 3). There was a slight increase in TNFα for CU#1 within 28 days. CU#1 had an elevation in CRP and IL-6 that was attributed to bacteremia from an infected vein port at day 4, 6, and 8. However, levels of CRP and IL-6 began to drop below baseline by day 14 through day 28. Additionally, D-Dimer concentration decreased in this patient on day 28. CU#2 also showed a decrease in CRP and IL-6 levels by day 14 through day 28, however, TNFα and D-Dimer remained elevated. CU#3 showed high levels of inflammatory markers TNFα, IL-6, and D-Dimer up to day 8, however, declines in all markers were reported by day 21. Furthermore, CRP levels in CU#3 declined dramatically by day 21. IL-2 levels were below the detection level in all patients at all-time points.

**Table 3:** Inflammatory biomarkers.

**Patient**	**D-Dimer (ng/mL)**	**CRP(mg/dL)**	**IL6 (pg/mL)**	**IL2(pg/mL)**	**TNFα (pg/mL)**
CU#1	Day 0: 1,033	Day 0: 2.66	Day 0: 19.39	Day 0: <38.0	Day 0: 0.95
	Day 4: 1,113	Day 4: >8.00	Day 4: 50.83	Day 4: <38.0	Day 4: 2.06
	Day 6: 1,069	Day 6: >8.00	Day 6: 51.95	Day 6: <38.0	Day 6: 2.08
	Day 8: 1,165	Day 8: >8.00	Day 8: 40.29	Day 8: <38.0	Day 8: 2.29
	Day 14: 1,122	Day 14: 1.28	Day 14: 8.23	Day 14: <38.0	Day 14: 2.58
	Day 21: 963	Day 21: 0.93	Day 21: 7.70	Day 21: <38.0	Day 21: 2.97
	Day 28: 599	Day 28: 0.70	Day 28: 6.43	Day 28: <38.0	Day 28: 1.36
CU#2	Day 0: 5,871	Day 0: 1.74	Day 0: 23.62	Day 0: <38.0	Day 0: 1.77
	Day 4: 4,305	Day 4: 1.79	Day 4: 22.17	Day 4: <38.0	Day 4: 1.99
	Day 6: 2,377	Day 6: 2.49	Day 6: –	Day 6: –	Day 6: –
	Day 8: 1,980	Day 8: 4.19	Day 8: 32.82	Day 8: <38.0	Day 8: 1.69
	Day 14: 3,444	Day 14: 1.04	Day 14: 17.79	Day 14: <38.0	Day 14: 2.13
	Day 21: 3,627	Day 21: 0.59	Day 21: 16.57	Day 21: <38.0	Day 21: 1.90
	Day 28: 4,468	Day 28: 0.35	Day 28: 13.58	Day 28: <38.0	Day 28: 2.21
CU#3	Day 0: 1,098	Day 0: 6.48	Day 0: 29.78	Day 0: <38.0	Day 0: 7.85
	Day 4: 1,130	Day 4: 2.15	Day 4: 28.71	Day 4: <38.0	Day 4: 6.51
	Day 6: –	Day 6: –	Day 6: –	Day 6: –	Day 6: –
	Day 8: 1,082	Day 8: 1.98	Day 8: 1,259	Day 8: <38.0	Day 8: >10
	Day 14: 1,647	Day 14: 2.79	Day 14: 7.9	Day 14: <38.0	Day 14: 6.53
	Day 21: 828	Day 21: 0.18	Day 21: 7.12	Day 21: <38.0	Day 21: 4.67
	Day 28: –	Day 28: –	Day 28: –	Day 28: –	Day 28: –

–*, sample not taken*.

## Discussion

These completed case studies are the first demonstrations of human amniotic fluid-derived nanoparticles as a safe and potentially efficacious therapeutic treatment to recover from complications induced by COVID-19 infection. The multi-dose administration of Zofin as a therapeutic approach for patients severely ill from COVID-19 was safe and well-tolerated, without the report of any serious adverse events. The molecular composition of Zofin, particularly the nanoparticle population that includes perinatal secreted extracellular vesicles and exosomes, has strong potential as a COVID-19 therapeutic (19). Extracellular and exosome-based therapeutics are beginning to be explored in the clinic and have quickly emerged as a promising therapeutic candidate due to the anti-inflammatory and tissue regenerative effects shown across various pre-clinical models (20–22). For example, the delivery of exosomal cargo to recipient macrophages stimulates M2 polarization that leads to the reduction of pro-inflammatory cytokine secretion (23). Similarly, exosome-mediated transfer of miRNA between immune cells may contribute to immune response at various cellular pathway levels, such as the suppression of pro-inflammatory response initiated in the presence of endotoxins (24). Based on this pre-clinical data, it is hypothesized that the immune modulatory effect of exosomes is particularly useful in mitigating symptoms associated with COVID-19 infection, and to promote the induction of endogenous tissue repair (25).

In these three cases, we are limited by the absence of experimental controls. First, the absence of a placebo control group and administration of the therapy as open label does not allow for scientific proof of efficacy. Similarly, the combination of ongoing care with Zofin treatment makes it difficult to determine the extent of therapeutic efficacy derived solely from the therapeutic. Therefore, the precise therapeutic value of Zofin can only be speculated based on the collected data post-treatment. With these limitations in mind, the primary take-away from these single patient cases is focused on safety, accessibility, and feasibility. As a new biologic, the primary objective of small, single patient studies is to demonstrate therapeutic safety. Safety was strictly monitored during product infusion and throughout the following days by the treating physician and onsite nurses. There was no reported appearance of any adverse reactions or adverse events. Therefore, Zofin was determined to be safe for the treated patients. Furthermore, these first completed studies support the accessibility and feasibility of the therapeutic. Zofin is an acellular biologic that requires minimal training and specialized equipment to ship, prepare for infusion, and administration. The product can be stored in standard medical freezers (below −20°C) and can be prepared for infusion by the onsite hospital pharmacist. There were no issues with drug handling and preparation at the hospital site. This experience is an important step toward the development of a therapy with wide-spread distribution potential and rapid incorporation into clinics.

Despite the experimental limitations, analysis of the collective data in all patients showed a reduction of SOFA score, improvement in ICU clinical status, and respiratory improvements. To date, the patient's laboratory results have shown improvements with decreased inflammatory biomarkers. Because of the small patient population and the lack of a placebo control, it is premature to determine the potential mechanism of action for the observed clinical effects. However, inflammatory biomarkers CRP and IL-6 decreased in all patients, similarly to other reported cases utilizing cell therapies, thus allowing for the further investigation of an anti-inflammatory effect (26, 27). All data was reported by the treating physician and nursing staff. Patients were not asked to share their perspective of the treatment outcome.

The clinical features of patient CU#1 improved considerably with lungs improving on CXR and both mental status and kidney function returning to normal. Respiratory function of this patient improved 21 days post-treatment, transitioning from a 21% T-collar to room air PMV and decannulation on day 26, representing a considerable achievement for this patient demographic. Inflammatory marker status of this patient, IL6 and CRP, improved after the 14-day time point. The patient further improved to hospital discharge after 29 days post-treatment initiation.

The clinical features of patient CU#2 systemically improved, including respiratory function, during the treatment time course. The patient transitioned from CPAP 5 PS 10 30% ventilation to 30% T-Collar ventilation by day 28 and the acute delirium improved. This patient sustained acute kidney injury and required regular hemodialysis during the study period.

After receiving Zofin, patient CU#3 displayed rapid improvement in respiratory function, with a complete decannulation from oxygen therapy by day 4. The patient had a complete recovery of renal function, had decreased creatinine concentration levels, and was removed from hemodialysis by day 17. CU#3 was discharged 26 days post-treatment initiation.

Our positive experience with these three patients further warrants placebo-controlled testing to determine the therapeutic effects of Zofin in this patient population. Organicell Regenerative Medicine is currently conducting the first multicenter, randomized, double-blinded, placebo-controlled phase I/II clinical trial to test Zofin in COVID-19 patients with moderate to severe Acute Respiratory Distress Syndrome.

## Data Availability Statement

The original contributions presented in the study are included in the article/supplementary materials, further inquiries can be directed to the corresponding author/s.

## Ethics Statement

The studies involving human participants were reviewed and approved by letters of acknowledgment by Independent Review Board-Western Institutional Review Board. The patients/participants provided their written informed consent to participate in this study. Written informed consent was obtained from the individual(s) for the publication of any potentially identifiable images or data included in this article.

## Author Contributions

MM: study design, data interpretation, writing, figures. MB: study design, data interpretation, writing, data analysis, literature search, figures. AS and MS: patient care, data collection, data interpretation. WK and AM: study design. KV: data collection. GH: data collection, data analysis, figures, writing. DS: study design, writing, product manufacturing. ZA, IS, and JM: study design, product manufacturing. GS: study design, data analysis, data interpretation, writing. All authors contributed to the article and approved the submitted version.

## Conflict of Interest

MM, AM, and GS reports personal fees and other from Organicell Regenerative Medicine, Inc., outside the submitted work. In addition, MM has a patent COMPOSITIONS COMPRISING NANOPARTICLES, METHOD OF MAKING AND USES THEREOF pending. MB, GH, DS, ZA, IS, and JM report personal fees from Organicell Regenerative Medicine, Inc., outside the submitted work. WK is a medical advisor to Organicell Regenerative Medicine. The remaining authors declare that the research was conducted in the absence of any commercial or financial relationships that could be construed as a potential conflict of interest.
